# Cytological evaluation, culture and genomics to evaluate the microbiome in healthy rabbit external ear canals

**DOI:** 10.1111/vde.13256

**Published:** 2024-05-14

**Authors:** Nikoleta Makri, Natalie Ring, Darren J. Shaw, Athinodoros Athinodorou, Victoria Robinson, Gavin K. Paterson, Jenna Richardson, Debbie Gow, Tim Nuttall

**Affiliations:** ^1^ Royal (Dick) School of Veterinary Studies and the Roslin Institute University of Edinburgh Midlothian UK; ^2^ Present address: Veterinary Specialists Scotland Livingston UK

**Keywords:** Otic cytological evaluation, ear commensal, ear culture, microbiome, metagenomics, ear anatomy

## Abstract

**Background:**

Lop‐eared rabbits may be predisposed to otitis externa (OE) as a consequence of their ear conformation. Although otoscopy, otic cytological evaluation and culture are valuable tools in dogs and cats, published data on rabbits remain lacking.

**Hypothesis/Objectives:**

This study aimed to assess the utility of otoscopy and cytological results in evaluating healthy rabbit external ear canals (EECs) and to characterise ear cytological and microbiological findings through culture techniques and metagenomic sequencing.

**Animals:**

Sixty‐three otitis‐free client‐owned rabbits.

**Materials and Methods:**

All rabbits underwent otoscopy and ear cytological evaluation. In a subset of 12 rabbits, further bacterial and fungal culture, fungal DNA assessment and metagenomic sequencing were performed.

**Results:**

Otic cytological results revealed yeast in 73%, cocci in 42.9% and rods in 28.6% of healthy rabbit EECs. Compared to upright‐eared rabbits, lop‐eared rabbits had more discharge and more bacteria per oil immersion field. Culture isolated eight different species yet metagenomic sequencing identified 36, belonging to the Bacillota (Firmicutes), Pseudomonadota and Actinomycetota phyla. *Staphylococcus* were the most commonly observed species with both methods. Ten of 12 rabbits were yeast‐positive on cytological evaluation with only three yielding fungal growth identified as *Yarrowia* (*Candida*) *lipolytica*, *Eurotium echinulatum* and *Cystofilobasidium infirmominiatum*.

**Conclusions and Clinical Relevance:**

Healthy rabbit EECs lack inflammatory cells yet can host yeast and bacteria, emphasising the need to evaluate cytological results alongside the clinical signs. Lop‐ear anatomy may predispose to bacterial overgrowth and OE. Notably, yeasts may be present despite a negative culture.

## Graphical abstract

**Background** – Lop‐eared rabbits may be predisposed to otitis externa (OE) as a consequence of their ear conformation. Although otoscopy, otic cytological evaluation, and culture are valuable tools in dogs and cats, published data on rabbits remain lacking. **Hypothesis/Objectives** – This study aimed to assess the utility of otoscopy and cytological results in evaluating healthy rabbit external ear canals (EECs), and to characterise ear cytological and microbiological findings through culture techniques and metagenomic sequencing. **Conclusions and Clinical Relevance** – Healthy rabbit EECs lack inflammatory cells yet can host yeast and bacteria, emphasising the need to evaluate cytological results alongside the clinical signs. Lop‐ear anatomy may predispose to bacterial overgrowth and OE. Notably, yeasts may be present despite a negative culture.

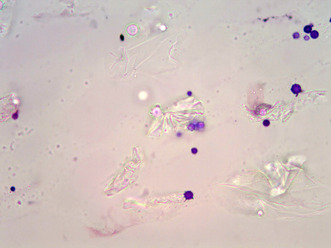

## Zusammenfassung

**Hintergrund:**

Kaninchen mit Hängeohren könnten eine Prädisposition zur Otitis externa (OE) als Konsequenz ihrer Ohrkonformation haben. Obwohl Otoskopie, zytologische Evaluierung des Ohres und Kultur gute Untersuchungsmöglichkeiten bei Hunden und Katzen darstellen, fehlt es an publizierten Daten für Kaninchen.

**Hypothese/Ziele:**

Diese Studie zielt darauf ab, die Anwendbarkeit von Otoskopie und zytologischen Ergebnissen bei der Evaluierung des externen Ohrkanals gesunder Kaninchen (EECs) zu untersuchen und die zytologischen und mikrobiologischen Befunde mittels Kulturtechniken und metagenomischer Sequenzierung zu charakterisieren.

**Tiere:**

Dreiundsechzig Kaninchen in Privatbesitz ohne Otitis.

**Materialien und Methoden:**

Bei allen Kaninchen wurde eine otoskopische und eine zytologische Untersuchung der Ohren durchgeführt. In einer Untergruppe von 12 Kaninchen wurden zusätzlich eine bakteriologische und eine mykologische Kultur sowie eine Untersuchung auf Pilz DNA und metagenomische Sequenzierung gemacht.

**Ergebnisse:**

Bei der zytologischen Untersuchung der Ohren wurden Hefepilze bei 73%, Kokken bei 42,9% und Stäbchen bei 28,6% der EECs der gesunden Kaninchen gefunden. Im Vergleich zu Kaninchen mit aufstehenden Ohren hatten die Kaninchen mit Hängeohren vermehrt Absonderungen und mehr Bakterien pro Ölimmersionsfeld. Mittels Kultur wurden acht verschiedene Spezies identifiziert, während mittels metagenomischer Sequenzierung 36 identifiziert wurden, die zu den Bacillota (Firmicutis), Pseudomonadota und Actinemycetota Stämmen gehörten. *Staphylococcus* war bei beiden Methoden die am häufigsten gefundene Spezies. Zehn von 12 Kaninchen waren zytologisch Hefepilze‐positiv, während nur bei drei ein Pilzwachstum in Form von *Yarrowia* (*Candida*) *lipolytica*, *Eurotium echinulatum* und *Cystofilobasidium infirmominiatum* erfolgte.

**Schlussfolgerungen und klinische Bedeutung:**

Die EECs gesunder Kaninchen haben keine Entzündungszellen gezeigt, können aber Hefepilze und Bakterien beherbergen, was die Bedeutung der gleichzeitigen zytologischen Evaluierung begleitend zu den klinischen Zeichen unterstreicht. Die Anatomie der Hängeohren könnte zu bakteriellem Überwachstum und OE prädisponieren. Vor allem ist zu bemerken, dass Hefepilze trotz einer negativen Kultur auftreten können.

## 摘要

**背景:**

垂耳兔可能因其耳朵形态而易患外耳炎。尽管耳镜检查、耳细胞学评估和培养是犬和猫的宝贵工具，但关于兔子的公开数据仍然缺乏。

**假设/目的:**

本研究旨在评估耳镜检查和细胞学结果在评估健康兔外耳道(EECs)中的作用，并通过培养技术和宏基因组测序来表征耳细胞学和微生物学结果。

**动物:**

63只无耳炎的客户饲养的兔子。

**材料和方法:**

所有兔均接受耳镜检查和耳细胞学检查。在12只兔子的子集中，进行了进一步的细菌和真菌培养、真菌DNA评估和宏基因组测序。

**结果:**

耳细胞学结果显示，健康兔EECs酵母为73%，球菌为42.9%，杆状菌为28.6%。与直耳兔相比，垂耳兔的每个油镜视野和细菌数量更多。培养分离出8个不同的菌种，但宏基因组测序确定出了36种，属于厚壁菌门、假单胞菌门和放线菌门。葡萄球菌是两种观察方法中最常见的菌种。12只兔子中有10只在细胞学评估中呈酵母菌阳性，只有3只出现真菌生长，被鉴定为解脂耶氏(念珠菌)酵母菌、冠突散囊菌和脆红泡囊线黑粉菌。

**结论和临床相关性:**

健康兔EECs无炎症细胞，但能容纳酵母菌和细菌，强调需要在评估临床症状的同时评估细胞学结果。垂耳兔耳朵解剖结构可能容易导致细菌过度生长和OE。值得注意的是，尽管培养物为阴性，酵母菌仍可能存在。

## Résumé

**Contexte:**

Les lapins béliers peuvent être prédisposés aux otites externes (OE) en raison de la conformation de leurs oreilles. Bien que l'otoscopie, l'évaluation cytologique auriculaire et la culture soient des outils précieux chez les chiens et les chats, il n'existe pas de données publiées chez le lapin.

**Hypothèse/Objectifs:**

Cette étude vise à évaluer l'utilité de l'otoscopie et de la cytologie dans l'évaluation des conduits auditifs externes (CAE) de lapins sains, et à caractériser les résultats cytologiques et microbiologiques de l'oreille par des techniques de culture et de séquençage métagénomique.

**Animaux:**

Soixante‐trois lapins exempts d'otite et appartenant à des clients.

**Matériels et méthodes:**

Tous les lapins subissent un examen otoscopique et une évaluation cytologique auriculaire. Dans un sous‐ensemble de 12 lapins, des cultures bactériennes et fongiques, une évaluation de l'ADN fongique et un séquençage métagénomique sont réalisés.

**Résultats:**

Les examens cytologiques auriculaires révèlent la présence de levures dans 73%, de cocci dans 42.9% et de bâtonnets dans 28.6% des CAE de lapins sains. Par comparaison aux lapins à oreilles dressées, les lapins béliers ont plus d'écoulements et plus de bactéries par champ dans l'huile à immersion. La culture permet d'isoler huit espèces différentes, mais le séquençage métagénomique en identifie 36, appartenant aux phyla *Bacillota* (Firmicutes), *Pseudomonadota* et *Actinomycetota*. Les staphylocoques constituent les espèces les plus fréquemment observées avec les deux méthodes. L'évaluation cytologique révèle la présence de levures chez dix des douze lapins, dont trois seulement présentent une croissance fongique identifiée comme *Yarrowia* (Candida*) lipolytica, Eurotium echinulatum* et *Cystofilobasidium infirmominiatum*.

**Conclusions et pertinence clinique:**

Les CAE de lapins sains sont dépourvus de cellules inflammatoires mais peuvent héberger des levures et des bactéries, ce qui souligne la nécessité d'évaluer les résultats cytologiques simultanément avec les signes cliniques. L'anatomie des oreilles de lapin peut prédisposer à la prolifération bactérienne et à l'OE. Et notamment, des levures peuvent être présentes malgré une culture négative.

## 要約

**背景:**

ロップイヤー・ラビットは、その耳の形状の結果として外耳炎(OE)になりやすい可能性がある。犬や猫では耳鏡検査、耳細胞学的評価、培養は貴重な手段であるが、ウサギに関する発表データはまだ不足している。

**仮説/目的:**

本研究の目的は、健常ウサギの外耳道(EECs)を評価する際の耳鏡検査および細胞学的結果の有用性を評価し、培養技術およびメタゲノム配列決定によって耳の細胞学的および微生物学的所見の特徴を明らかにすることであった。

**対象動物:**

耳炎のないオーナー所有ウサギ63匹。

**材料と方法:**

すべてのウサギに耳鏡検査および耳細胞学的評価を行った。12羽のウサギのサブセットでは、さらに細菌および真菌の培養、真菌DNA評価、メタゲノム配列決定を行った。

**結果:**

耳細胞診の結果、健常ウサギのEECの73%に酵母、42.9%に球菌、28.6%に桿菌が認められた。直立耳ウサギと比較して、ロップイヤー・ラビットは分泌物が多く、油浸野あたりの細菌数も多かった。培養により8種が分離されたが、メタゲノム配列決定により、バシラス門(ファーミキューテス門)、シュードモナス門、放線菌門に属する36種が同定された。どちらの方法でもブドウ球菌が最も多く観察された。12羽のウサギのうち10羽が細胞学的評価で酵母陽性を示し、真菌の増殖が認められたのは*Yarrowia* (*Candida*) *lipolytica*、Eurotium *echinulatum*、*Cystofilobasidium infirmominiatum*のみであった。

**結論と臨床的関連性:**

健常ウサギのEECは炎症細胞を欠くが、酵母や細菌を宿主とする可能性があることから、臨床症状とともに細胞診結果を評価する必要性が強調された。ロップイヤーの解剖学的構造は、細菌の過剰増殖やOEの素因となる可能性があった。注目すべきは、培養が陰性であるにもかかわらず酵母が存在することであった。

## Resumo

**Contexto:**

Coelhos de orelhas caídas podem ser predispostos à otite externa (OE) como consequência desta conformação anatômica. Apesar da otoscopia, avaliação citológica otológica e cultura serem ferramentas úteis em cães e gatos, faltam dados publicados em coelhos.

**Hipótese/Objetivos:**

Este estudo teve como objetivo avaliar a utilidade da otoscopia e dos resultados citológicos na avaliação de orelhas externas (EECs) saudáveis de coelhos, e caracterizar os achados citológicos e microbiológicos através de técnicas de cultura e sequenciamento metagenômico.

**Animais:**

Sessenta e três coelhos de clientes sem otite.

**Materiais e métodos:**

Todos os coelhos foram submetidos à otoscopia e avaliação citológica. Em um grupo de 12 coelhos, realizou‐se cultura fúngica e bacteriana, avaliação de DNA fúngico e sequenciamento metagenômico.

**Resultados:**

Os resultados de citologia otológica revelaram leveduras em 73%, cocos em 42,9% e bastonetes em 28,6% dos EECs de coelhos saudáveis. Comparados aos coelhos de orelhas eretas, os coelhos de orelhas caídas tiveram mais secreção e mais bactérias por campo de imersão. A cultura isolou oito espécies diferentes, mas o sequenciamento metagenômico identificou 36, pertencentes aos filos Bacillota (Firmicutes), Pseudomonadota e Actinomycetota. *Staphylococcus* foram as espécies mais comumente observadas com ambos os métodos. Dez dos 12 coelhos foram positivos para leveduras na avaliação citológica, mas apenas três apresentaram crescimento de espécies fúngicas que foram identificadas como *Yarrowia (Candida*) *lipolytica*, Eurotium echinulatum e *Cystofilobasidium infirmomiiatum.*

**Resultados:**

Os EECs de coelhos saudáveis não possuem células inflamatórias, mas podem ser hospedeiros de leveduras e bactérias, enfatizando a necessidade de avaliar os resultados citológicos juntamente com os sinais clínicos. A anatomia das orelhas caídas pode predispor ao supercrescimento bacteriano e à OE. Notavelmente, leveduras podem estar presentes apesar de uma cultura negativa.

## RESUMEN

**Introducción:**

Los conejos de orejas caídas pueden estar predispuestos a sufrir otitis externa (OE) como consecuencia de la conformación de sus orejas. Aunque la otoscopia, la evaluación citológica ótica y el cultivo son herramientas valiosas en perros y gatos, aún faltan datos publicados sobre conejos.

**Hipótesis/Objetivos:**

Este estudio tuvo como objetivo evaluar la utilidad de la otoscopia y los resultados citológicos en la evaluación de los conductos auditivos externos (EECs) de conejos sanos, y caracterizar los hallazgos citológicos y microbiológicos del oído mediante técnicas de cultivo y secuenciación metagenómica.

**Animales:**

Sesenta y tres conejos de propietarios particulares libres de otitis.

**Materiales y Métodos:**

A todos los conejos se les realizó otoscopia y evaluación citológica del oído. En un subconjunto de 12 conejos, se realizaron más cultivos bacterianos y fúngicos, evaluación del DNA fúngico y secuenciación metagenómica.

**Resultados:**

Los resultados citológicos óticos revelaron levaduras en el 73%, cocos en el 42,9% y bacilos en el 28,6% de los EECs de conejos sanos. En comparación con los conejos de orejas erguidas, los conejos de orejas caídas tenían más secreciones y más bacterias por campo de inmersión en aceite. El cultivo aisló ocho especies diferentes pero la secuenciación metagenómica identificó 36, pertenecientes a los filos *Bacillota* (*Firmicutes*), *Pseudomonadota* y *Actinomycetota*. Los estafilococos fueron las especies más comúnmente observadas con ambos métodos. Diez de 12 conejos dieron positivo a levaduras en la evaluación citológica y solo tres produjeron crecimiento fúngico identificado como *Yarrowia* (*Candida*) *lipolytica*, *Eurotium echinulatum* y *Cystofilobasidium infirmominiatum*.

**Conclusiones y relevancia clínica:**

Los EECs de conejos sanos carecen de células inflamatorias, pero pueden albergar levaduras y bacterias, lo que enfatiza la necesidad de evaluar los resultados citológicos junto con los signos clínicos. La anatomía de la oreja caída puede predisponer al crecimiento excesivo de bacterias y a la OE. En particular, pueden estar presentes levaduras a pesar de un cultivo negativo.

## INTRODUCTION

Rabbits are the third most common pet in the UK, with an estimated population of 1.1 million.^1^ Otitis externa (OE) is considered common, although estimates of the prevalence vary. A UK survey found that 21.2% of rabbits had veterinary‐diagnosed ear issues,^2^ while a larger US study reported that only 3.5% had OE.^3^ Lop‐eared rabbits may be predisposed to OE^4^; a survey reported ear disease in 25% of lop‐eared versus 10% in upright‐eared rabbits.^2^

In dogs and cats, otoscopy and cytological evaluation are cost‐effective ways to assess the external ear canal (EEC) and select appropriate treatments.^5^, ^6^ However, knowing the cytological results, culture and microbiota of healthy ears is necessary to interpret samples from rabbits with OE, and thereby enhance treatment selection and antimicrobial stewardship.

A previous study on otic cytological results of healthy dwarf rabbits found that 54% had yeast (which were likely to be *Malassezia* spp.), with 80% having <5 yeast cells/oil immersion field (OIF), although otoscopy was not evaluated.^7^ Another study reported yeasts in 20 of 30 rabbits, with subjectively more discharge in lop‐eared versus upright‐eared rabbits.^4^ Bacteria were rare in both studies, with no significant differences between lop‐ and upright‐eared rabbits; however, neither study performed quantitative assessments or sequencing.^4^, ^7^

Two studies investigating the otic mycobiota could not culture *Malassezia* spp. despite cytological evidence of yeast.^8^, ^9^ Ultimately *Malassezia* phylotype 131^8^ and *M. cuniculi* ^9^ were identified via next‐generation sequencing (NGS) of DNA. Conversely, in a recent US study utilising NGS, *Cladosporium* was the most abundant species in both affected and healthy ears. However, these results were not correlated with cytological results or otoscopy.^10^

The limited data on healthy rabbit ears hinder interpretation of the findings in rabbits with OE. Therefore, this study aimed to (a) determine the utility of otoscopy and cytological results in evaluating EECs in healthy rabbits, and (b) characterise EEC cytological and microbiological findings using culture and metagenomic sequencing. We hypothesised that rabbits have a diverse otic microbiome that can be mapped using their cytological results, facilitating prompt diagnosis and treatment decisions.

## MATERIALS AND METHODS

### Ethics

Ethical approval was granted by the University of Edinburgh Veterinary Ethical Review Committee (125.21). Rabbit owners gave informed consent before enrolment.

### Study population

This was a single‐centre prospective study. All rabbits were presented to the Exotic Animal Service at the Royal (Dick) School of Veterinary Studies Hospital for Small Animals between February 2022 and April 2023. They were examined by a specialist or resident in rabbit and exotic pet medicine, and by a resident in veterinary dermatology. The inclusion criteria were as follows: systemically healthy, no prior history of ear disease, and no topical ear treatments or oral antibiotics within the previous three months. Rabbits with skin lesions, ear disease and/or that did not tolerate otoscopic examination were excluded. The reason for the presentation, the breed, sex, age, bodyweight and lifestyle (indoor/outdoor), any in‐contact animals and ear anatomy (lop/upright) were recorded. Any treatments following the aural examination were noted. Patient anonymity was ensured by using assigned case numbers.

### Data collection and sampling

Otoscopy, cytological and microscopic evaluation were performed in all rabbits. As a consequence of time and funding constraints, only the last subset of 12 rabbits had cultures and DNA sequencing performed. The samples were collected by a dermatological or an exotic pet medicine resident and transported to the microbiology and genomics laboratories within three hours and processed on the same day.

### Otoscopy

Standardised otoscopy was performed in both EECs of each rabbit using a veterinary hand‐held otoscope (Heine G100) with metal, cold sterilised otoscope cones. Blinded scores for exudate type and amount were recorded by two investigators using a subjective score formulated for this study (see Figure S1). If visible, the clinical appearance of the tympanic membrane (TM) was recorded; if not visible, the reason for this was recorded.

### Cytological and microscopical evaluation

A single nonsterile cotton swab was gently rotated in the wall of the base of each EEC to obtain a cytological sample. Care was taken to avoid inserting the swab into the blind ending pouch rostral to the tragus (the entrance to the EEC is caudal to the tragus in rabbits).^11^ The sample was rolled on a microscopic slide in a standardised manner,^12^ stained with a modified Wright's stain (Diff‐Quik; Atom Scientific), rinsed in tap water and air‐dried. Another sample, mounted in liquid paraffin with an overlying cover slip, was examined for ectoparasites (e.g. *Psoroptes cuniculi*) immediately after collection on low magnification (×40). All slides were examined microscopically (Olympus BX41) by the same investigator.

**FIGURE 1:**
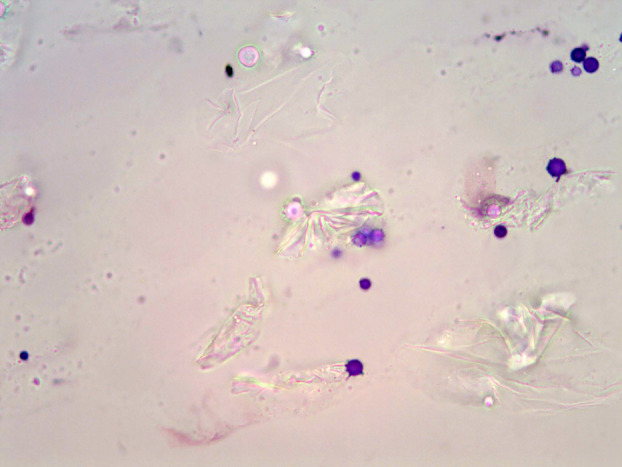
Smear from an ear swab (Rabbit 18) showing the presence of yeast cells. Diff‐Quik stain, ×1000.

The slides were initially scanned on ×100 magnification to identify representative areas. Bacteria appeared symmetrical, smooth‐walled and usually uniformly stained, with sizes varying between 1 and 2 μm depending on their morphology (rod/cocci). Yeasts typically surrounded squamous cell clumps, and were round, singular or occasionally budding and ranged in size from 3 × 4 to 4 × 7 μm. They were often arranged in clusters and not always uniformly distributed (Figure 1). The total number of inflammatory cells, yeast, cocci and rods was recorded across 10 representative ×1000 OIFs. A second blinded microscopy was conducted by the same investigator for repeatability assessment. In this study, we employ the term microbes to refer to cocci, rods and yeast.

### Bacterial/fungal culture, internal transcribed spacer (ITS) sequence analysis

One sterile swab (charcoal) was collected for standard culture from a single ear of each rabbit using simple random sampling (coin toss—head: right, tails: left). Samples were collected before the cytological swabs were taken to avoid contamination. Swabs were cultured overnight (16–18 h) at 37°C aerobically on Columbia blood agar with 5% (v/v) horse blood and MacConkey agar (E&O Laboratories Ltd) with an additional blood plate incubated anaerobically (16–18 h at 37°C). In the case of no growth, plates were incubated for a further 24 h. Bacterial identification and antimicrobial susceptibility testing (using Clinical and Laboratory Standards Institute/CLSI criteria Vet 2017 V8.02 based on M100.S25^13^) were performed using the Vitek2 (BioMérieux) The same swab was utilised for fungal culture using Sabouraud dextrose agar with chloramphenicol (SabC) and potato dextrose agar (PDA) in light and dark room conditions and at 37°C for five days. Fungal isolates were identified by partial ribosomal ITS sequencing with the primers ITS1 and ITS4.^14^

### Microbiome swab enrichment, DNA extraction and metagenomic sequencing

An additonal ESwab (Copan) was collected from the ear of each of the rabbits in the subset of 12. A test extraction indicated extremely low cell loads precluding direct metagenomic extraction for amplification‐free whole genome sequencing. Instead, a ‘culturomics’ enrichment‐based strategy was employed.^15^, ^16^ Each ESwab was vortexed for 1–2 min, and then, 0.5 mL of the liquid medium was used to inoculate 60 mL each of Mueller–Hinton broth (MHB; Oxoid, Thermo Fisher Scientific) and Tryptone Soya Broth (TSB; Oxoid). Half of each broth was incubated in aerobic conditions and anaerobic conditions (AnaeroGen sachets; Oxoid). All samples were incubated for 72 h at 37°C with shaking. The same volume of uninoculated broth was incubated under the same conditions as a negative control. After incubation, DNA was extracted using the MagAttract HMW DNA kit (Qiagen) using MetaPolyzyme (3.3 mg/mL; Sigma‐Aldrich) for metagenomic cell lysis (see Methods S1 for full methods). MetaPolyzyme includes two enzymes (chitinase and lyticase) which lyse the fungal cell wall, and therefore, most fungi present, if any, should have been lysed allowing their DNA extraction. The DNA samples were sequenced on a GridION using the Native Barcoding Kit 24 v14 and two MinION R10.4.1 flow cells (kit LSK‐NBD114.24; Oxford Nanopore Technologies) according to manufacturer's instructions (Methods S1; Table S1).

### Data analysis

Real‐time super‐accurate (SUP) guppy (v6.5.7) base‐calling was carried out on the GridION during the sequencing runs. Sequencing read quality control was carried out using nanoplot (v1.40.0)^17^; then, adapter and barcode sequences were trimmed using porechop (v0.2.4).^18^ The trimmed reads were assembled using flye with the ‐‐meta and ‐‐nano‐hq options (2.9.1),^19^ and the draft assemblies were polished using medaka (v1.7.2; https://github.com/nanoporetech/medaka) with the raw reads and r1041_e82_400bps_sup_g615 model.

Next, the polished contigs were binned using metabat2 (v2.2.15),^20^ and checkm (v1.0.13)^21^ was used to check the quality of each resulting bin (i.e. to determine if each bin contained only one metagenome assembled genome [MAG]). Each MAG was visualised using bandage.^22^ The most likely bacterial species was assigned to each MAG bin by performing a blastn search of the ‘nt_prok’ NCBI database (downloaded 31.08.2023). abricate (v1.0.1) (https://github.com/tseemann/abricate) was used to identify potential AMR genes in each MAG. Finally, the MAGs were scanned for fungal species using a blastn search of the ‘28S_fungal_sequences’ NCBI database (downloaded 31.08.2023).

### Statistical methods

Statistical analysis was performed using minitab 2.1 (LLC State College). Data were assessed using the Anderson–Darling test for normality. Non‐normal data were presented as the median, interquartile range (IQR) and range, and subject to Mann–Whitney statistical tests. Categorical variables were summarised as counts and percentages and subject to chi‐square or Fisher's exact tests (if the expected count in any cell was <5). Univariate analyses were performed between the cytological findings (median [yeast, cocci and rods]/OIF, presence of yeast/cocci/rods [yes/no]), median exudate scores and variables (sex, age, ear conformation, indoor/outdoor and quantity of exudate) using Spearman's correlation and binary logistic regression. McNemar and Wilcoxon signed‐rank paired tests were used to assess repeatability of the presence and the number of microbes/OIF, respectively. Statistical significance (α) was set at *p* ≤ 0.05.

## RESULTS

### Signalment

This study included 63 rabbits: 37 (58.7%) males (25 neutered) and 26 (41.3%) females (20 neutered) aged from 0.3 to 9.8 years (mean 4.4 years, SD 2.4 years). Only five rabbits were less than one‐year‐old. Median body weight was 2.33 kg (range 0.8–6 kg, interquartile range [IQR] 1.04 kg). Twenty‐four (38%) were cross‐breeds, and 39 (62%) were pure‐breds. Thirty‐two (51%) had lop ears, and 31 (49%) had upright ears. Thirty‐nine (61.9%) were housed indoors only. All of the rabbits were systemically healthy, and most (66.6%) were presented for routine vaccination or health checks between March and August 2022 and 2024 (46 of 63; 73%). Most owners had other pets (50 of 63; 79.4%), and most of these were other rabbits (46 of 50; 92%). The subset for further studies included six lop‐ and six upright‐eared rabbits (six male, six female) with a median age of three years (range 0.7–9.8 years, IQR 6.2 years) and a mean body weight of 2.4 kg (SD 0.63 kg). Nine were indoor‐only, and eight were pure‐bred.

### Otoscopy

All rabbits tolerated otoscopic examination well and none were excluded through an inability to examine the ears or distress during the procedure. The TM was visualised in one or both ears in 23 of 63 rabbits (36.5%); visualisation was possible in over half of the upright‐eared rabbits (18/31; 58.1%) and fewer lop‐eared rabbits (five of 32; 15.6%) (*p* < 0.05). No ear mites were seen. Lop‐eared rabbits were significantly (*p* < 0.001) more likely to have ear exudate scores more than zero (29 of 32; 90.6%) (Table 1) compared to upright‐eared rabbits (10 of 31; 32.2%), 28.1% of lop‐eared rabbits had an exudate score ≥2 compared to none of the upright‐eared rabbits (Fisher's exact test; *p* < 0.05).

**TABLE 1:** Rabbits (% [n]) with positive external ear canal (EEC) cytological results for yeast, cocci, rods and all microbes (yeast or cocci or rods) examined in 10 oil immersion fields (OIF; ×1000).

Ear conformation	Yeast**	Cocci*	Rods*	All microbe*	Exudate*
Upright (n = 31)	64.5% (20/31)	29% (9/31)	16.1% (5/31)	80.7% (25/31)	32.3% (10/31)
Lop (n = 32)	81.3% (26/32)	56.3% (18/32)	40.6% (13/32)	100% (32/32)	90.6% (29/32)
All rabbits	73% (46/63)	42.9% (27/63)	28.6% (18/63)	90.5% (57/63)	61.9% (39/63)

*Note*: The last column shows rabbits with average exudate score >0 on otoscopy. Significance: *, *p* < 0.05; **, *p* > 0.05.

### Cytological results

Yeasts were detected in 46/63 (73%; 95% confidence interval (CI) 60.4, 83.4), cocci in 27/63 rabbits (42.9%; CI 30.5, 55.9) and rods in 18/63 (28.6%; CI 17.9, 41.3). Lop‐eared rabbits were more likely to have positive cytology for microbes (overall *p* = 0.009, and *p* < 0.037 for cocci and rods individually) (Table 1). More lop‐eared rabbits had yeast (81%) compared to upright‐eared rabbits (65%), but this was not statistically significant (*p* = 0.14) (Table 1). Six rabbits (9.5%) had no microbes. No ectoparasites or inflammatory cells were seen.

There was a positive association between the presence or absence of rods and cocci (*p* < 0.001), but not between the presence of cocci/yeast or rods/yeast (*p* > 0.054). There were statistically significant associations between the presence of yeast, cocci and rods with sex (*p* < 0.029), with fewer males positive for yeast (62%) compared to females (89%), and more males positive for cocci (60%) and rods (41%) than females (19% and 12%, respectively). Neutered rabbits were more likely to have yeasts in their ears and not cocci/rods. There were no associations of age, indoor/outdoor lifestyles or exudate with the presence of yeast, cocci and rods (*p* > 0.085).

**FIGURE 2:**
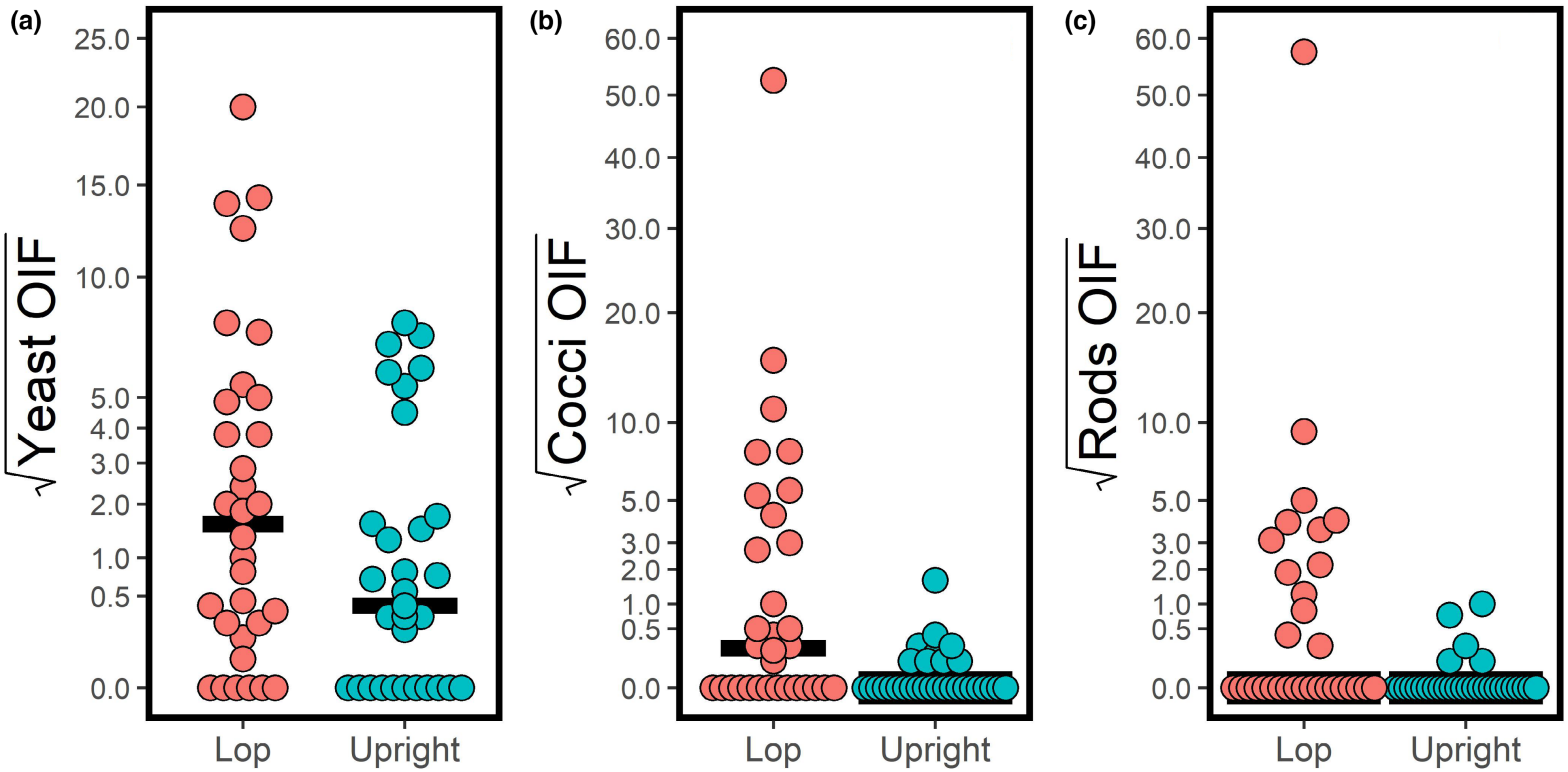
Individual value plot showing square‐root transformed number of yeast (a), cocci (b) and rods (c) per oil immersion field (OIF) in external ear canal (EEC) cytological results of 32 lop‐eared versus 31 upright‐eared healthy rabbits. Solid black lines are the median levels for the two groups of rabbits.

Most rabbits (49 of 63; 77.8%) had <5 yeasts/OIF. Only four (6.3%; all lop‐eared) had >10 yeast/OIF. Lop‐eared rabbits had higher median yeast/OIF counts than upright‐eared rabbits, but this was not statistically significant (*p* = 0.10) (Table 2; Figure 2). Lop‐eared rabbits did, however, have statistically significantly more cocci (*p* = 0.003) and rods (*p* = 0.011) than upright‐eared rabbits (Table 2; Figure 2). Age and lifestyle (indoor/outdoor) were not significantly associated with the numbers of microbes (*p* > 0.11). By contrast, cocci were more frequent in male (median/OIF = 0.1) than female rabbits (median/OIF = 0) (*p* = 0.003), yet there was no difference for yeast (*p* = 0.125). The median value for rods was the same in both sexes (0; with 23 of 26 females and 22 of 37 males having no rods). In those rabbits with rods, the rods were more frequent in males (range 0.1–57.5) than females (range 0.4–4) (*p* = 0.013). Neutered rabbits had higher median yeast/OIF counts (1.5) than entire rabbits (0.08), and there was no association of neuter status with the numbers of cocci or rods. There was no association between the amount of exudate and numbers of yeast or rods (*p* > 0.192), yet there was an association with cocci (*ρ* = 0.251, *p* = 0.047).

**TABLE 2:** Median number and range of yeast, cocci and rods seen in external ear canal (EEC) cytological results of healthy rabbits counted on 10 oil immersion fields (OIF; ×1000).

Micro‐organism	Ears (U/L)	Median/OIF	Range
Yeast**	U	0.4	0–7.9
	L	1.6	0–20
	All	1.9	0–20
Cocci*	U	0	0–1.7
	L	0.2	0–52.5
	All	0.1	0–52.5
Rods*	U	0	0–1
	L	0	0–57.5
	All	0	0–57.5

*Note*: Significance: *, *p* < 0.05; **, *p* > 0.05.

Abbreviations: L, lop; U, upright.

Microscopic assessments by the same investigator showed no difference in the presence and counts of cocci/rods per OIF. A significant difference was observed for presence and counts of yeast (*p* < 0.003) with the first evaluation not corresponding with the second in seven cases.

Kruskal–Wallis tests revealed no differences in the number of cocci (*p* = 0.53), rods (*p* = 0.38) and yeast (*p* = 0.48) between the subset of 12 rabbits and the remaining 51 rabbits. We therefore assumed that these were a representative sample for further microbial culture and metagenomics.

### Culture

Culture from nine of 12 rabbits was positive for bacteria and/or yeasts. Eight different bacterial species were isolated by aerobic/anaerobic culture. These belonged to the phyla Bacillota, Pseudomonadota and Actinomycetota (Table 3). The most frequently observed were *Staphylococcus* spp. in four of nine, with *S. xylosus* the most commonly isolated, followed by *Aeromonas salmonicida* in two of nine. In one rabbit, a meticillin‐resistant strain of *S. xylosus* was isolated although no cocci were observed on cytological evaluation.

**TABLE 3:** Comparison of cytological results, culture and sequencing of micro‐organism in 12 rabbits.

Rabbit	Ear (U/L)	Ear sampled (coin toss)	Yeast	Cocci	Rods	Culture Vitek/ITS	Metagenomic sequencing
1	L	Left	Yes	No	No	**Moraxella group** Gram‐stain‐negative coccus (unidentified)	***Corynebacterium glutamicum*** *Staphylococcus* spp. *Staphylococcus equorum* *Enterococcus faecium* *Staphylococcus xylosus* *Aerococcus viridans or urinaeequi* *Staphylococcus succinus*
2	U	Left	Yes	No	No	Negative	*Rothia* spp. *Psychrobacter* spp. *Staphylococcus succinus* *Aerococcus viridans* or *urinaeequi* *Staphylococcus caeli* *Staphylococcus equorum* ***Bacillus licheniformis***
3	U	Left	Yes	No	No	Negative	*Mammaliicoccus vitulinus* ***Lelliottia amnigena***
4	U	Right	Yes	Yes	No	*Staphylococcus equorum* ^a^ *Staphylococcus capitis* * Eurotium echinulatum *	*Staphylococcus equorum* ^a^ *Streptococcus* spp. *Aerococcus viridans or urinaeequi* *Staphylococcus ureilyticus or cohnii* ***Bacillus velezensis* or *Bacillus amyloliquefaciens***
5	L	Left	Yes	No	No	Negative	*Mammaliicoccus vitulinus* *Staphylococcus equorum* *Staphylococcus chromogenes* ***Lactobacillus* spp.** *Staphylococcus saprophyticus* *Aerococcus viridans* or *urinaeequi*
6	L	Right	Yes	No	No	*Kocuria rosea* *Staphylococcus xylosus* ^b^	*Staphylococcus hominis* *Micrococcus luteus* *Staphylococcus shinii* *Staphylococcus capitis* *Staphylococcus* spp.
7	L	Right	Yes	No	No	***Pantoea agglomerans*** * Candida lipolytica *	***Bacillus pumilus*** ***Bacillus licheniformus*** ***Acinetobacter lwoffii*** *Staphylococcus equorum* *Streptococcus suis or Streptococcus pneumoniae*
8	L	Right	Yes	Yes	No	*Staphylococcus xylosus* ***Sphingomonas paucimobilis*** ***Aeromonas salmonicida***	***Leclercia* sp.** ***Enterococcus durans*** *Staphylococcus haemolyticus* ***Leucobacter muris*** *Staphylococcus xylosus* ***Corynebacterium* spp.**
9	L	Right	Yes	Yes	Yes	***Aeromonas salmonicida***	Metagenomic assembly failed
10	U	Left	Yes	No	No	*Cystofilobasidium* spp. (likely * Cystofilobasidium infirmominiatum *)	*Streptococcus salivarius* ***Pantoea agglomerans*** *Streptococcus* spp. *Staphylococcus caeli* *Mammaliicoccus vitulinus*
11	U	Left	No	No	No	*Staphylococcus xylosus* ^a^ * Geomyces pannorum *	*Staphylococcus equorum* *Staphylococcus xylosus* ^a^ *Streptococcus* spp. ***Lysinibacillus fusiformis*** *Staphylococcus* spp. ***Bacillus licheniformis*** *Staphylococcus haemolyticus* *Staphylococcus equorum*
12	U	Left	No	No	Yes	* Eurotium amstelodami *	*Streptococcus ferus* ***Bacillus cereus*** ***Pseudomonas* spp.** *Staphylococcus epidermidis* *Rothia* spp. *Staphylococcus epidermidis/warneri*

*Note*: Distinction of microbe type: cocci, roman, rods, bold; yeasts, underlined.

Abbreviations: ITS, internal; L, lop; U, upright.

^a^ Culture (+)/next‐generation sequencing (NGS) (+).

^b^ Meticillin‐resistant.

Five fungi genera were isolated. These belonged to the phyla Ascomycota (*Eurotium echinulatum*, *Eurotium amstelodami*, *Geomyces pannorum*, *Yarrowia lipolytica*) and Basidiomycota (*Cystofilobasidium infirmominiatum*). Ten of 12 rabbits had yeast on cytological evaluation yet only three yielded fungal growth (*Yarrowia* [*Candida*] *lipolytica*, *Eurotium echinulatum* and *Cystofilobasidium infirmominiatum*). In two samples with no cytological evidence of fungal elements, *E. amstelodami* and *G. pannorum* were identified (respectively).

### GridION sequencing

All sequencing data has been deposited in the NCBI's Sequencr Read Archive under BioProject accesion PRJNA1109608 and SRA accessions SRR28975594 to SRR28975605. Using a culturomics approach, 36 bacterial species from three different phyla (Bacillota [Firmicutes], Pseudomonadota and Actinomycetota) were isolated. Metagenome assembly failed for one sample for unknown reasons (Table 3). The species range was 0–8 with a median of six species per rabbit ear. There were no phyla exclusively detected in lop‐eared or upright‐eared rabbits. Overall, 16 genera were identified: *Acinetobacter*, *Corynebacterium*, *Enterococcus*, *Lactobacillus*, *Leclercia*, *Leucobacter*, *Micrococcus*, *Aerococcus*, *Bacillus*, *Lysinibacillus*, *Mammaliicoccus*, *Pantoea*, *Staphylococcus*, *Streptococcus*, *Lelliottia*, *Pseudomonas*, *Psychrobacter* and *Rothia*. One genus was not determined owing to low homology with sequences deposited in public databases. Metagenomic sequencing identified AMR genes in all samples and did not identify the meticillin‐resistant *Staphylococcus xylosus* isolated on the culture of Rabbit 6 (Table 3; Table S2). *Staphylococcus* spp. was detected in all except one sample with successful genome assembly; *Staphylococcus equorum* was the most common. *Bacillus* spp. was isolated from five of 12 rabbits and *Aerococcus* spp. (*A. viridans* or *A. urinaeequi*) from four of 12. No fungi were identified by this approach, and there was no growth in the negative control broths.

## DISCUSSION

Ear disease has a significant impact on domestic rabbits and their owners.^2^, ^23^, ^24^ This is the first study evaluating the otoscopic, cytological, microbiological and microbiome data in a population of client‐owned healthy domestic rabbits.

In our study, yeast was found in 73% of rabbit ears suggesting that these are common commensals. This is higher than in previous reports where yeast was found in 66.7%^7^ and 54%.^4^ These findings are comparable to dogs (96%) and cats (83%).^25^ Contrary to previous studies (6.8%,^7^ 16%^4^), we identified cocci more commonly (42.9%) in healthy rabbits. This again is similar to dogs and cats where low numbers of cocci were found in 42% and 71%, respectively.^25^ Surprisingly, rods were found in 28.6% of the rabbits. This is notably different to previous reports in rabbits, dogs and cats where rods were absent.^4^, ^7^, ^25^ Furthermore, there was a positive correlation between the presence of rods and cocci, which implies that healthy rabbits may have a more mixed population of otic bacteria. These findings may be a consequence of biophysiological differences in small mammals, including higher body temperature,^26^ and distinct skin structure and physiology.^27^

Lop‐eared rabbits were more likely to have bacteria on cytological evaluation and had higher exudate scores, which correlated with increased cocci counts. Lop‐eared rabbit EECs are commonly stenosed from the level of the tragus, leading to accumulation of desquamated cells and cerumen.^11^, ^28^ The unique lop conformation and otic microenvironment is likely to predispose to bacterial dysbiosis,^2^, ^4^ although ear conformation was not associated in a similar way with the presence of yeast.^4^, ^7^ Discrepancies with previous rabbit studies may come from varying proportions of lop‐ and upright‐eared rabbits, sample size, purpose (pet versus breeding/meat), location, living conditions and contact with other animals. Our stringent inclusion criteria, particularly regarding oral medication (especially antibiotics), may also have influenced the results.

To the best of our knowledge, this is the first study quantifying microbes in cytological samples from healthy rabbit ears. The median yeast/OIF count was 1.9, which is much higher than those in dogs and cats.^25^, ^29^ Approximately 78% of the rabbits had <5 yeast/OIF, which is similar to previous findings,^7^ with much lower numbers of cocci and rods. No inflammatory cells were present, consistent with earlier rabbit,^7^ cat^30^ and dog^25^ studies. Therefore, the presence of inflammatory cells is likely to be associated with clinical OE in rabbits.

Assessing the TM was harder than anticipated, with only 36.5% visualised. This is likely to be to the result of variations in ear canal shape and size (especially dwarf breeds)^11^ and/or the presence of exudate particularly in lop‐eared rabbits. This emphasises the need for skilled clinicians and specialised equipment such as smaller otoscope heads for dwarf rabbits. Morphological differences hinder evaluation of the ear canal and eardrum (e.g. these were visualised in only 15.5% of lop rabbits), potentially making it harder to spot early signs of otitis.^31^ Additionally, lop rabbits may show a higher pain response during ear examination than upright rabbits do, affecting tolerance of otoscopy and ear canal evaluation.^4^ However, pain levels were not assessed in our study as these were healthy rabbits.

In order to characterise the yeast and bacteria found on cytological evaluation, 12 rabbits underwent further culture and metagenomic sequencing. Cytological results and culture generally lacked correlation, with seven of 10 cases with yeast in cytological results showing no fungal growth in culture (Table 3). Two other studies had similar results, although DNA extraction and sequencing ultimately identified *Malassezia* phylotype 131 and *M. cuniculi*.^8^, ^9^ Conversely, a recent large study comparing the ear canal microbiome in 34 healthy and 16 rabbits with otitis found that healthy rabbits had a more diverse microbiome with *Cladosporium* spp. being most prevalent, suggesting an association between *Malassezia* spp. and otitis.^10^ The cultured fungi, analysed by ITS1 and ITS4, belonged in the phyla Ascomycota and Basidiomycota, consistent with previous studies.^9^, ^10^ However, only 3 of 10 yeast‐positive samples produced fungal colonies, with none identified as *Malassezia* or *Cladosporium* spp., possibly as a consequence of slow‐growing/fastidious micro‐organisms or the media used. Despite swift processing in our and other studies, disproportional micro‐organism growth during transit cannot be entirely dismissed. In addition, small mammals' higher body temperature might necessitate incubation temperatures exceeding 37°C. This underscores the necessity for culture‐independent techniques.^32^

Bacterial culture revealed eight species whereas metagenomic sequencing found 36, with Pseudomonadota, Bacillota and Actinomycetota the dominant phyla. Only two of 12 rabbits had the same bacteria identified by both culture and NGS (*Staphylococcus equorum* and *S. xylosus*) (Table 3). In 6 of 12 samples, culture detected bacteria that NGS did not, yet NGS consistently identified more species, including in three culture‐negative cases. Under‐representation of facultative anaerobic bacteria (*Aerococcus* and *Bacillus* spp.) in culture may stem from anaerobic handling challenges. Slow‐growing bacteria such as *Corynebacterium* spp., identified solely by NGS in two samples, may be swamped by fast‐growing bacteria. In addition, as micro‐organism recovery varies between swab types,^33^ using charcoal swabs for culture and ESwabs for NGS may have influenced the outcomes.^34^ Using different media for culture and culturomics before NGS sequencing may also have favoured different species. Standardising parameters for both methods could mitigate inconsistencies. Culture‐independent methods offer accuracy, yet low biomass studies (such as rabbit ears) pose challenges.^35^ Metagenomic sequencing directly from the swabs was not possible owing to the extremely low biomass prompting a culturomics approach,^15^, ^16^, ^36^ which could have lost some bacterial or fungal species. Future studies may benefit from a culture‐independent approach, such as direct metagenomic DNA extraction from swabs followed by whole genome amplification (WGA). However, WGA also risks bias as some species' DNA amplify more efficiently than others.^37^

To the best of our knowledge this is the first study using nanopore sequencing to evaluate the microbes on rabbit skin. This identifies a greater array of bacteria compared to conventional techniques and allows study of the carriage and distribution of AMR genes. A small study on rabbit microbiota showed that Bacillota (Firmicutes) (62.37%), Proteobacteria (13.44%) and Bacteroidota (11.84%) were the predominant phyla with sparse information annotated at the species level.^38^ In Vecere et al. 2022, *Staphylococcus epidermidis* exhibited high relative abundance in healthy rabbit ears, while *Pseudomonas aeruginosa* was present only in otitis samples. In our study, a *Pseudomonas* sp. was isolated from an upright‐eared rabbit that had rods on cytology but was culture‐negative. *Staphylococcus* was the most common genus in both culture and GridION sequencing, indicating that they are important otic commensals. The *Staphylococcus xylosus*, *S. equorum*, *S. epidermidis* and *S. saprophyticus* isolated in our study are commonly found in wild rabbits,^39^ whereas *S. caeli* is a novel species recently discovered through air sampling in an Italian industrial rabbit facility.^40^ These findings highlight the potential influence of the environment (e.g. interaction with wild rabbits and pet/breeding facilities^8^) on the composition of rabbit otic flora. *Rothia* spp. and *Streptococcus* spp. are oral commensals in healthy rabbits.^41^ One sample yielded no significant hits in the NCBI nucleotide prokaryote database, suggesting a novel species. However, as we did not isolate this species no further identification or characterisation was possible. The presence and prevalence of AMR genes in pet rabbits has not been well‐studied,^42^, ^43^ making it unclear if the high prevalence of AMR that we found is common. It is important to note though, that gene presence does not always ensure phenotype expression with species‐specific correlation varying (e.g. 99% for *Staphylococcus aureus* and *E. coli*).^44^, ^45^

Our study has several limitations. We used a subjective otic exudate scoring system (score 0–3) adapted from previous published scales^4^; this lacks validation similar to that in dogs^46^ but this was beyond the scope of this project. Our results may not be applicable to different geographical regions, warmer climates, outdoor‐only rabbits or those in industrial settings.^8^ The division of the lop EEC into two compartments (i.e. proximal and distal to the fold) limits the usefulness of otoscopy and cytological evaluation. The intraobserver variability in cytological yeast counts may be a consequence of clustering and uneven distribution, and an alternative method may be needed to improve consistency. Future studies should also involve a second observer to assess interobserver variability. Funding constraints limited the number of rabbits for culture and metagenomics, although analysis indicated that these were representative of the whole group. Quantifying bacterial isolates and comparing bacterial counts or species based on ear conformation was unfeasible. NGS, potentially owing to the broth used, could not isolate fungi, precluding a comparison with culture results, and having a positive fungal control would have been beneficial for quality assurance and method validation.

In conclusion, conscious otoscopy and cytological evaluation were practical and well‐tolerated diagnostic tools. However, ear anatomy, especially in lop‐eared rabbits, may greatly compromise visualisation of the tympanic membrane, reducing the utility of this technique. Rabbit EECs can harbour a variety of yeasts and bacteria as normal commensals, highlighting the importance of assessing cytological results alongside clinical signs. Additionally, there is a probable association between the presence of inflammatory cells and clinical OE in rabbits. Lop‐eared rabbits appear predisposed to increased otic bacterial loads and cerumen accumulation. Establishing the normal ear flora is an initial step, yet further studies comparing cytological results in healthy and diseased rabbit ears are crucial. Such studies would enhance the qualitative and quantitative characterisation of the rabbit ear microbiome, aiding in treatment selection and disease monitoring.

## AUTHOR CONTRIBUTIONS

**Nikoleta Makri:** Methodology; writing – original draft; funding acquisition; formal analysis; data curation; visualization; investigation; validation; writing – review and editing. **Natalie Ring:** Writing – review and editing; data curation; methodology; investigation; visualization; software; validation. **Darren J. Shaw:** Formal analysis; writing – review and editing; data curation; methodology; visualization; software; validation. **Athinodoros Athinodorou:** Writing – review and editing; investigation. **Victoria Robinson:** Writing – review and editing; visualization; supervision; project administration; funding acquisition; conceptualization. **Gavin K. Paterson:** Writing – review and editing; investigation; methodology. **Jenna Richardson:** Writing – review and editing; methodology; investigation. **Debbie Gow:** Conceptualization; methodology. **Tim Nuttall:** Writing – review and editing; supervision; project administration; funding acquisition; methodology; visualization; conceptualization; validation.

## CONFLICT OF INTEREST STATEMENT

The authors declared no potential conflicts of interest with respect to the research, authorship, and/or publication of this article.

## Supporting information


Appendix S1.
