# Supplementary material for: Cytological evaluation, culture and genomics to evaluate the microbiome in healthy rabbit external ear canals
Source: Vet Dermatol. 2024 May 14;35(5):479–91. doi: 10.1111/vde.13256 (PMC11656672; doi:10.1111/vde.13256)
Supplement: Supplementary file 1 — Appendix S1. [file VDE-35-479-s001.docx]

**FIGURE S1.** ATLAS and exudate scoring

| **Grade** | **Quantity of cerumen** |
| --- | --- |
| 0 | None |
| 1 | Small |
| 2 | Moderate |
| 3 | Large |

{0} None


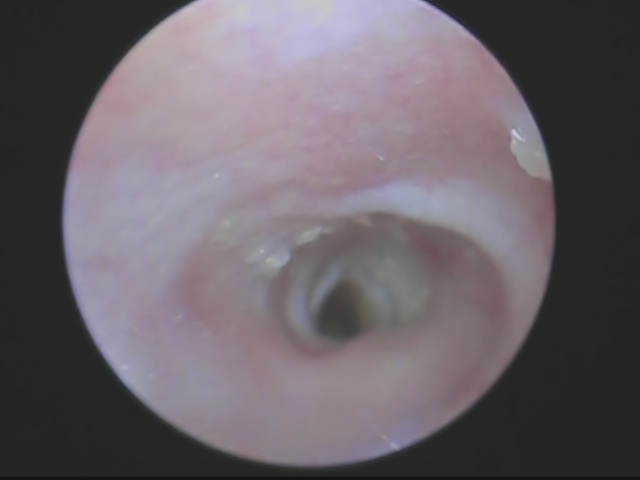


{1} Very small amounts of exudate visible in the ear canal


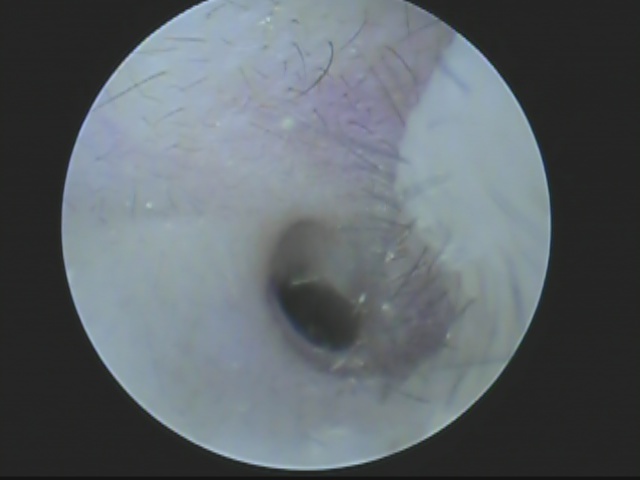


{2} Moderate amounts of exudate visible partially obscuring visualisation


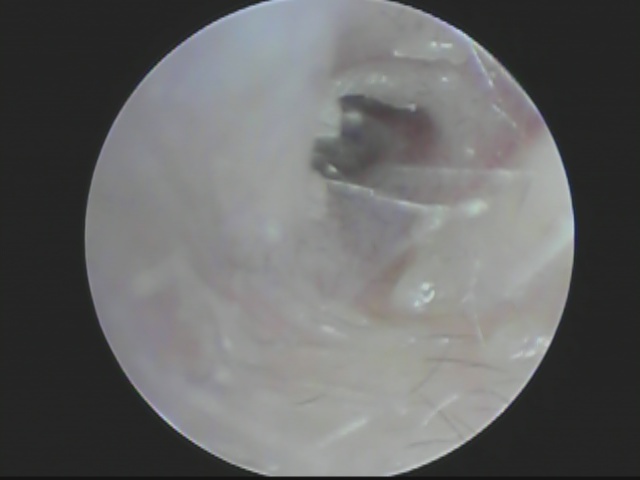


{3} Large amounts of exudate fully obscuring visualisation


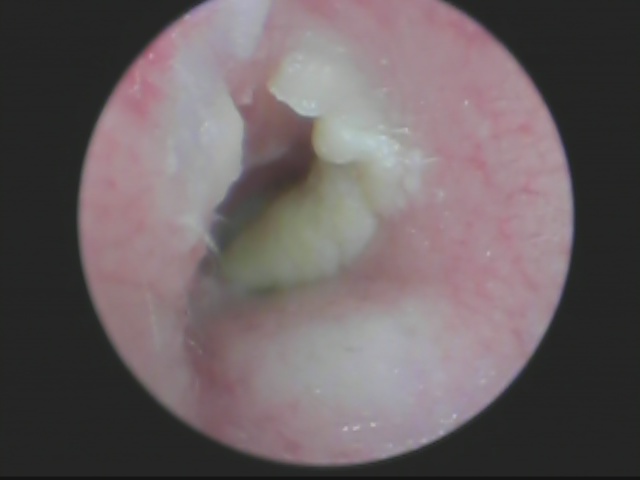


**METHODS S1. Microbiome swab enrichment, DNA extraction and metagenomic sequencing**

After 72 h of incubation, DNA was metagenomically extracted using the MagAttract HMW DNA kit (Qiagen). One millilitre of aerobically-grown Mueller–Hinton broth (MHB) was pooled with 1 mL aerobically-grown TSB, and 1 mL anaerobically-grown MHB was pooled with 1 mL anaerobically-grown TSB, producing two 2 mL aliquots. These were pelleted for 3 min at 16,000***g***, the supernatant discarded, and the pellets resuspended in 160 µL 50 mm Tris, 10 mm EDTA, pH 8.0 (buffer P1 as per the MagAttract HMW DNA protocol). To perform metagenomic bacterial lysis, 20 µL Metapolyzyme (3.3 mg/mL; Sigma-Aldrich) was added and mixed by flicking. The samples were incubated on a thermomixer for 3 h at 37 ^o^C with 900 rpm shaking, then the rest of the MagAttract HMW DNA protocol was followed as per the manufacturer’s instructions (from step 8 on p26 of the Handbook 03/2020), eluting into 200 µL nuclease-free water. The DNA concentration of each sample was quantified using the Qubit dsDNA HS kit as per the manufacturer’s instructions. The DNA samples were sequenced on a GridION using the Native Barcoding Kit 24 v14 and two MinION R10.4.1 flow cells (kit LSK-NBD114.24; Oxford Nanopore Technologies). Sequencing libraries were prepared using the Native Barcoding Kit 24 v14 (kit LSK-NBD114.24; Oxford Nanopore Technologies) according to the manufacturer’s instructions, starting with 750–1,000 ng DNA and using barcodes 1 to 7. The prepared libraries were sequenced on a GridION with two R10.4.1 (FLO-MIN114) flow cells performing real-time super-accurate (SUP) Guppy (v6.5.7) base-calling.

**TABLE S1.** Sequencing yields and read lengths of the 12 rabbit ear samples and

two negative controls

| Rabbit | Sequencing run | Barcode | Sequencing yield (Gbp) | Read length N50 (bp) |
| --- | --- | --- | --- | --- |
| 1 | 1 | 1 | 2.118 | 4,792 |
| 2 | 1 | 2 | 2.011 | 4,200 |
| 3 | 1 | 3 | 1.167 | 8,949 |
| 4 | 1 | 4 | 2.044 | 3,918 |
| 5 | 1 | 5 | 2.697 | 2,511 |
| 6 | 1 | 6 | 1.693 | 3,631 |
| Negative1 | 1 | 7 | 0.002 | 5,276 |
| 7 | 2 | 1 | 1.624 | 11,728 |
| 8 | 2 | 2 | 2.088 | 10,686 |
| 9 | 2 | 3 | 0.872 | 12,003 |
| 10 | 2 | 4 | 2.845 | 5,908 |
| 11 | 2 | 5 | 3.045 | 5,434 |
| 12 | 2 | 6 | 3.855 | 5,856 |
| Negative2 | 2 | 7 | 0.005 | 5,323 |

**TABLE S2.** Antimicrobial resistance (AMR) genes found in metagenomic sequencing

| **Rabbit** | **Species predicted** | **AMR gene detected in species** |
| --- | --- | --- |
| 1 | *Enterococcus* strain 1 | tet(M) (tetracycline) |
|  | *Enterococcus* strain 2 | aac(6')-Ii (aminoglycoside), msr(C) (macrolide) |
|  | *Staphylococcus xylosus* | mph(C) (macrolide) |
|  | *Enterococcus* strain 3 | eat(A) (pleuromutilin) |
| 2 | *Bacillus licheniformus* | blaP (beta-lactam), rphC (rifamycin), erm(D) (macrolide) |
|  | *Aerococcus viridans*/*urinaeequi* | tet(M) (tetracycline) |
|  | *Staphylococcus caeli* | mecI_of_mecC, mecC3 (methicillin), blaZ-mecC (beta-lactam) |
|  | *Staphylococcus equorum* | mph(C) (macrolide) |
| 3 | *Mammaliicoccus vitulinus* | mecA2 (methicillin) |
|  | *Lelliottia amnigena* | oqxB15, oqxA6 (phenicol, quinolone) |
| 4 | *Staphylococcus equorum* | mph(C) (macrolide) |
|  | *Staphylococcus ureilyticus*/*cohnii* | fusF (fusidic acid) |
|  | *Bacillus velezensis*/*amyloliquefaciens* | satA_Bs (streptotricin), rphC (rifamycin), clbA (lincosamide, macrolide, streptogramin) |
| 5 | *Lactobacillus* spp. | tet(W) (tetracycline) |
|  | *Aerococcus viridans*/*urinaeequi* | dfrG (trimethoprim) |
| 6 | *Staphylococcus hominis* | mph(C) (macrolide) |
|  | *Staphylococcus capitis* | blaZ, blaR1, blaI_of_Z (beta-lactam) |
| 7 | *Bacillus pumilis* | blaBPU-1 (beta-lactam), cat86 (chloramphenicol) |
|  | *Bacillus licheniformus* | blaP (beta-lactam), rphC (rifamycin) |
|  | *Acinetobacter lwoffii* | blaOXA-335 (carbapenem) |
|  | Unknown 1 | tet(33) (tetracycline) |
|  | *Streptococcus suis*/*pneumoniae* | tet(W) (tetracycline) |
|  | Unknown 2 | tet(H) (tetracycline) |
| 8 | Unknown 1 | vanR-O (vancomycin) |
|  | *Leclercia* spp. | oqxB9 (phenicol/quinolone), oqxA10 (phenicol/quinolone) |
|  | *Enterococcus durans* | Aac(6’)-lih (aminoglycoside), tet(M) (tetracycline) |
|  | *Leclercia* spp. | fosA8 (fosfomycin) |
| 10 | *Streptococcus salivarius* | mef(A) (macrolide), msr(D) (macrolide), lsa(C) (lincosamide, streptogramin) |
|  | *Pantoea agglomerans* | oqxB11 (phenicol, quinolone) |
|  | *Staphylococcus caeli* | mecI_of_mecC, mecC3 (methicillin), blaZ-mecC (beta-lactam) |
| 11 | *Staphylococcus haemolyticus* | blaZ, blaR1, blaI_of_Z (beta-lactam), mph(C), msr(A) (macrolide) |
| 12 | *Streptococcus ferus* | tet(O) (tetracycline), lsa(C) (lincosamide, streptogramin) |
|  | *Bacillus cereus* | vanZ-F (vancomycin), fosB (fosfomycin), bla1 (beta-lactam), Bcll (carbapenem) |
|  | *Staphylococcus epidermidis* | dfr(C) (trimethoprim), fusB (fusidic acid), fosB (fosfomycin), erm(C) (macrolide) |
|  | *Staphylococcus epidermidis*/*warneri* | fusF (fusidic acid) |
